# Categorical Nature of Major Factor Selection via Information Theoretic Measurements

**DOI:** 10.3390/e23121684

**Published:** 2021-12-15

**Authors:** Ting-Li Chen, Elizabeth P. Chou, Hsieh Fushing

**Affiliations:** 1 Institute of Statistical Science, Academia Sinica, Taipei 11529, Taiwan; tlchen@stat.sinica.edu.tw; 2 Department of Statistics, National Chengchi University, Taipei 11605, Taiwan; eptchou@g.nccu.edu.tw; 3 Department of Statistics, University of California, Davis, CA 95616, USA

**Keywords:** CEDA, conditional entropy, conditional mutual information, heterogeneity, information gain

## Abstract

Without assuming any functional or distributional structure, we select collections of major factors embedded within response-versus-covariate (Re-Co) dynamics via selection criteria [C1: confirmable] and [C2: irrepaceable], which are based on information theoretic measurements. The two criteria are constructed based on the computing paradigm called Categorical Exploratory Data Analysis (CEDA) and linked to Wiener–Granger causality. All the information theoretical measurements, including conditional mutual information and entropy, are evaluated through the contingency table platform, which primarily rests on the categorical nature within all involved features of any data types: quantitative or qualitative. Our selection task identifies one chief collection, together with several secondary collections of major factors of various orders underlying the targeted Re-Co dynamics. Each selected collection is checked with algorithmically computed reliability against the finite sample phenomenon, and so is each member’s major factor individually. The developments of our selection protocol are illustrated in detail through two experimental examples: a simple one and a complex one. We then apply this protocol on two data sets pertaining to two somewhat related but distinct pitching dynamics of two pitch types: slider and fastball. In particular, we refer to a specific Major League Baseball (MLB) pitcher and we consider data of multiple seasons.

## 1. Introduction

Two news articles have recently been published on the topic of Major League Baseball (MLB) pitchers’ performance being drastically empowered or caused by baseball’s spin rate increases. One is a FiveThirtyEight article published in 2019 and titled “How Gerrit Cole Went from So-So to Unbeatable?”, and the other is a 2021 New York Times article with the title, “Once Again, MLB Faces a Crisis of Its Own Making”. The connection between the two articles is the MLB pitcher Gerrit Cole. In fact, from the 2017 season to 2018 and 2019, his spin rate significantly increased, and this increase has been indicated as the cause of his drastically improved performance. In fact, it is known that MLB pitchers have been using various kinds of substances to enhance their grip on baseballs and consequently improve their performance. The New York Times article discusses the MLB’s ban of Foreign Substances—also called sticky substances—in June 2021, and confidently echoes that the increase in spin rate has indeed immensely improved MLB pitchers’ performance, which is seemingly unfair to all batters in MLB.

Have such cause-and-effect relationships been rigorously established based on MLB databases, such as PITCH/x and Statcast? The answer is likely negative. In fact, if we take a collection of pitches delivered by an MLB pitcher in a single season as a data set observed from a pitcher-specific pitching dynamics with complexity [1,2], then one fundamental problem called the Many System Problem (MSP) underlies both news articles. An MSP can be concisely depicted using the following question: How can one rigorously study a collection of somewhat similar but different complex dynamics or systems? Indeed, no such MSP has been well-studied and reported in the scientific literature yet.

A complex system can be intuitively characterized as a system composed of multiple interacting constituent mechanisms that generate macroscopic collective behaviors of temporal, spatial or functional structures. In other words, from all structural aspects, its whole is greater than the sum of its parts. This characteristic should naturally proclaim more discoveries to be found within each complex system in this Big Data era. Then, this [more data]-[more discoveries] phenomenon ideally should mirror what was described by Nobel laureate physicist P.W. Anderson [3] in his 1972 Science paper titled “More is Different”.

Is this proclamation of more discoveries also true for MSPs in general? The answer to this seemingly straightforward question is indeed complicated because of the heterogeneity of the involved complex systems. For example, the above two MLB-related MSPs’ temporal- and spatial-free functional complex systems are all governed by the same principles, but they are all somehow heterogeneously distinct. Such heterogeneity naturally induces multi-scale and hierarchical structures across all individual systems. Therefore, the complications in the answer to the above question are tied to a fundamental issue: How would such system heterogeneity impact the evaluation of key features pertaining to the shared principles in a collective fashion? We study the MSP consisting of three season-based systems in this paper, but defer the study of the MSP with many hundreds of systems to a separate report. In other words, in this paper, we primarily focus on computational developments for data-driven MSP study.

Our computational developments begin with treating a collection of complex systems as a meta-complex system with one categorical ID as one of its observable features. By keeping this aspect of the meta-complex system in mind, we see that the computational issue underlying the foundation of all MSPs can generically be stated as follows: How can one coherently study a system’s dynamics characterized partly or entirely by categorical features?

This fundamental computing issue is not classic, especially when the system under study is too complex to afford an analytical description, such as the pitching dynamics of an MLB pitcher. As such, the dynamics of interest are often depicted in terms of response-versus-covariate (Re-Co) dynamics, in which the response variable might involve multiple response features, not merely one, and the number of covariate features might be large. The categorical features could be on the response side as well as on the covariate side. Without an available analytic representation of such Re-Co dynamics, this fundamental issue becomes especially critical in this Big Data era, since no model is expected to be coherent within a big data setting. This fact is guaranteed by Anderson’s “More is Different” phenomenon [3].

Specifically speaking, in this paper, we computationally develop a resolution to this fundamental issue. Under the setting of imposing no assumed functional structure upon a targeted Re-Co dynamics that characterizes an MSP under study, we construct a selection protocol by employing information theoretic measures, such as conditional entropy and mutual information, to identify one chief collection and several alternative collections of major factors underlying the targeted Re-Co dynamics. Such a computational resolution in the form of a collection of major factors aims to realistically discover constituent parts or mechanisms pertaining to a complex system under study. Although this concept of major factors seems intuitive, the authors are not aware of any available discovery protocols in the literature. In fact, as will be seen below, the goal of discovering collections of major factors induces rather interesting fundamental computational complexity.

In order to avoid confusion in names, we remark that our major factor of complex dynamics is intrinsically distinct from the “factor” of the popular “factor analysis”, common in the psychology literature. Factor analysis has no Re-Co dynamics, and its computations rely entirely on Principle Component Analysis, which is primarily built on linearity and normality assumptions [4]. The resultant factors cannot offer direct and straightforward interpretations. In contrast, our data-driven major factors have a clear meaning and offer transparent interpretations, leading to the understanding of the complex systems under study.

In sharp contrast, our major factor selection protocol is developed based on a newly developed computational paradigm called Categorical Exploratory Data Analysis (CEDA) [5,6], which works for all structured data types: continuous, discrete and categorical. In other words, CEDA makes good use of the categorical nature contained in data types.

The rest of this paper is organized as follows. In Section 2, relevant background materials are reviewed and discussed. In Section 3, information theoretic measures are discussed, as well as our computational developments for the major factor selection protocol. They are illustrated and motivated through two experimental systems, a simple one and a complex one, with large simulated data sets. In Section 4, our selection protocol is applied to select multiple collections of major factors of Gerrit Cole’s slider and fastball pitching dynamics across the 2017, 2018 and 2019 seasons. Based on the computed collections of major factors of various orders, we are able to make a conclusion regarding the roles of spin rate in Cole’s slider and fastball pitching dynamics across the three MLB seasons.

## 2. Background

Baseball pitching dynamics is governed physically by the Magnus effect of aerodynamics and biomechanically by pitchers’ idiosyncratic pitching gesture [7]. The Magnus effect, a special version of Netwon’s second law of force, depicts how a spinning object travels through a medium, such as air. Though this aerodynamic principle is, in general, well-known in baseball pitching, its functional structures for each single pitch of one single pitcher could vary in many ways. In addition to the already complicated details of biomechanics exerted from pitching gesture, including horizontal (X−), vertical (Z−) and pitcher-to-catcher (Y−) directional forces (or accelerations), starting speeds and releasing point coordinates, the environmental conditions, such as wind speed and humidity in the stadium and the surface conditions of the baseball, all need to be taken into account. Hence, realistically building an analytical system of differential equations for the Magnus effect is a rather difficult task. As such, a complex system of pitching dynamics is practically impossible to acquire for each individual pitcher, and there are no grounds to pursue a unified analytical system description or model for one pitcher across multiple seasons. For this reason, this analytic direction and other model-based approaches are not pursued or even included for comparison in this paper.

Before our methodological developments, we very briefly depict all features involved in the common theme used in the MLB examples. The horizontal and vertical movements are denoted by $\left\{pfx_{x},pfx_{z}\right\}$, and horizontal (X−) and vertical (Z−) directional accelerations denoted as {aX,aZ}. Both pairs are designated 2D response variables, respectively, for the first and second phases of analysis. In contrast, the covariate features include the following: the pitcher-to-catcher directional acceleration aY; spin direction (spinD); spin rate (spinR); releasing point’s coordinates: x0, z0; three directional releasing speeds: vX0,vY0,vZ0; and, lastly, pitcher name (pitN). All response and covariate features are quantitative, except for the pitN, which is categorical.

As for the computational paradigm called Categorical Exploratory Data Analysis (CEDA), it has been recently developed [5,6]. Its fundamental idea is stated as follows: let all features’ natural categories assemble freely in order to shed light on the true pattern information contained in data. Though the name EDA was coined by John Tukey [8], the categorical nature-based CEDA with the above idea is fundamentally independent of the data analysis approaches and methodologies in Tukey’s works on EDA.

The first step of CEDA is to categorize each response and covariate feature via its histogram, which can be properly built via an effective algorithm developed in [9]. This step is to reduce noise embraced by all measurements in order to reveal its intrinsic categorical structure. The second step employs the contingency table for all developments involving information theoretic measures. It is used as a platform for coupling multiple categorized features together to form and define a new composite variable. This contingency table platform also serves as a platform for visualizing and evaluating possibly non-linear associations between any two variables. The two directional associations are numerically evaluated via conditional (Shannon) entropy. By properly re-scaling with respect to the corresponding marginal (Shannon) entropy, the mutual conditional entropy (MCE) [10] is calculated. This association measure is the appropriate one when handling categorical features.

The third step of CEDA is the focal point of this paper. To effectively depict this step with simplicity, we consider that each complex (meta-)system’s dynamics is coherently captured by a Re-Co association between possibly multiple response features, denoted by Y, and many 1D covariate features, denoted by $\left\{V_{k}|k=1,\ldots ,K\right\}$. The available structured data set is an ensemble of vectors: $\left(Y,V_{1},\ldots ,V_{K}\right)$. Each component feature of the data vector can be of any data type: continuous, discrete or categorical. All quantitative features would be categorized in the first step of CEDA. Since there exists no risk of confusion throughout this paper, all categorized features will retain their original notations.

In the second step of CEDA, the contingency tables would facilitate all marginal and conditional entropy evaluations. We propose to compute the conditional entropies of Y given all possible covariate feature combinations or feature sets, since features’ structural categories are allowed to reassemble freely upon all these response-versus-covariate contingency tables, without being subject to man-made constraints. As such, this collection of all possible directional associations ideally should contain all vital associative patterns that can indicate all constituent mechanisms underlying the designated Re-Co dynamics of Y against $\left\{V_{k}|k=1,\ldots ,K\right\}$ within an MSP. However, this collection of directional associations is a huge trove of information. It is likely too large to be properly processed without an effective feature selection protocol.

Since each of its categories of categorized Y can be taken as a label, we can seemingly treat the Re-Co dynamics of Y against $\left\{V_{k}|k=1,\ldots ,K\right\}$ as a classification problem. There exists extensive literature on feature selection under a classification problem setting [11,12] that employs information theoretic measures, such as mutual information and other variant entropies [13,14]. For instance, various popular filter versions of feature selection methods can generically be stated as means of finding an optimal feature subset $S^{*}\subseteq \left\{V_{k}|k=1,\ldots ,K\right\}$ such that $S^{*}$ achieves the optimal value with respect to various pre-determined goal functions. These typically consist of one relevancy term and one redundancy term [12,15]. The relevancy of feature subset $S\subseteq \{V_{k}|k=1,\ldots ,K\}$ is defined through joint mutual information between feature subsets *S* and Y, denoted as I[S;Y], and cardinalities |S|, while the redundancy of a feature $V_{k}$ is evaluated through marginal mutual information of $V_{k}$ and a selected feature subset *S*. The optimal feature subset is meant to balance between relevancy and redundancy in order to achieve the goal of minimizing the training time and maximizing the classification accuracy.

However, the goal of achieving classification accuracy is not equal to the goal of achieving the understanding of complex dynamics as a whole by discovering all its constituent parts. Furthermore, the concept of redundancy obviously misses the potential of interacting effects of multiple marginally independent features that are indeed conditionally dependent given Y. This conditional dependency given Y is one major construct of constituent parts or mechanisms embraced by a complex Re-Co dynamics. Therefore, it is clear that we need goals that are different from mere classification, and we need to consider the perspectives of discovering the true characteristics of a complex system.

In this paper, we attempt to identify all feature sets that correspondingly manifest all constituent mechanisms of the targeted Re-Co dynamics. Each mechanism-specific feature set is called a major factor. Our construction of a major factor selection protocol is designed to achieve this goal with reliability. This is the chief computational contribution of this study. We briefly elaborate on the concept of a major factor and our selection criteria here.

A major factor, say $A^{*}$, of Y is a subset of covariate features with an order being equal to its cardinality $|A^{*}|\left(=k\right)$. Intuitively speaking, an order-*k* major factor must contain the predictability of Y. Such predictability is visible upon a contingency table as the exact platform displaying a (covariate-category)-versus-(response-category) framework. For instance, let all categories of $A^{*}$ be arranged along the table’s row axis, and all categories of Y along its column axis. The predictability can be seen on a row-by-row basis and evaluated via row-wise Shannon entropy, which is specifically termed conditional entropy (CE) of Y given a category of $A^{*}$ [13,14]. It must be, in general, much smaller than the marginal Shannon entropy of Y calculated from the vector of column sums. In other words, the overall conditional entropy of Y given $A^{*}$ must achieve a significant drop. How large must a CE drop be in order to be claimed as significant? This seemingly simple issue indeed is complicated by the sample size at hand. We develop an algorithm for the first part of the necessary condition for $A^{*}$ to be declared as a major factor of Y with reliability.

The second part of the necessary condition is also obvious, but much more involved. If $A^{*}$’s order *k* is greater than 1, then $A^{*}$ is required to also achieve the so-called “ecological effect”. From the confirmation aspect, the ecological effect here specifically means that the CE drop achieved by $A^{*}$ as a whole must be significantly larger than the sum of its chief subset’s CE drop and many times its secondary subsets’ individual CE drops. Meanwhile, from the underlying dynamics perspective, member features of $A^{*}$ must form a unique and “irrepaceable” conditional dependence conditioning on Y. As we will demonstrate in the next section, this ecological effect indeed can be measured by feature members’ “conditional mutual information” given Y minus its marginal one. The third part of the necessary condition is that any identified major factor $A^{*}$ can couple with other covariate features to become a new higher-order major factor if its reliability check holds.

By satisfying the above three parts of the necessary conditions, if an identified major factor $A^{*}$ is also chronologically preceding the response variable Y, then $A^{*}$ is basically fulfilling the two components of Wiener–Granger causality mentioned in C. W. J. Granger’s 2003 Nobel Lecture [16]:

(1) the cause occurs before the effect; and
(2) the cause contains information about the effect that is unique, and is in no other variable.

This simple and concise concept of Wiener–Granger causality has been widely used in many scientific areas beyond economics, such as physiology [17], neuroscience [18] and meteorology [19], to name just a few. In the physics literature, the popular concept of information flow is closely linked to “causality”, when two systems’ dynamics are represented via two or two sets of time series, by using another entropy measure called transfer entropy, developed in [20]. This linkage has been popularly explored and used [21], and their equivalent relation has also been established [22,23]. Outside of causality, various definitions of entropy are also created and employed within many topics in physics—for instance, the intrinsic predictability of a time series concerning how to predict future values [24].

In this paper, we make use of information theoretical measures, but refrain from exploring causality. On the one hand, our computational developments aim at accommodating a universal setting whereby the response variable Y is simply represented by a completely unknown global function of multiple unknown mechanisms constituted by major factors of various orders. In other words, Y and all covariate features $\left\{V_{k}|k=1,\ldots ,K\right\}$ are not necessarily in chronological order. Without assuming any man-made structures, our focal issue of discovering major factors of various orders fundamentally does not align well with Wiener–Granger causality based on modeling, mathematical logic, graph theory, Bayesian probability, etc. [25].

On the other hand, the information theoretic measures, such as mutual information and conditional entropy, in fact match the data’s categorical nature well and have played key roles along CEDA developments from its beginning [5,6,10]. In the MLB example, we do not consider evolutions of pitching dynamics along the temporal axis here.

## 3. Methods

In this section, we first briefly review some popular concepts of information theoretic measures used in this study. Then, we illustrate our data-driven computational developments for our CEDA-based selection protocol for major factors. Such CEDA developments rely on the categorical nature of all involved features: response and covariate. We emphasize once more that the response variable Y possibly involves multiple features of any data types: continuous, discrete or categorical or their combination. If Y involves continuous or discrete features, each feature is categorized with respect to its own histograms [9]. For expositional simplicity and without concerns of notational ambiguity, we still use Y to denote its categorized version. All covariate features, denoted as $V_{k}$ with k=1,…,K, are either categorical or categorized 1D covariate features.

Here, we also make use of capital letters *A* or *B* to denote different subsets of covariate features when there is no need to specify their feature memberships. Based on the categorical nature of all the features, Y, *A* and *B* can be treated as 1D composite categorical variables with each occupied hypercube as a category. From the perspective of a 1D composite categorical variable, the information of original neighboring systems of categorized features is not entirely lost.

Any pair of 1D categorical features defines a contingency table, as do pairs of categorical variables such as (Y,A), (Y,B) and (A,B). Once a contingency table is available, information theoretic measurements are natural tools for discovering associative patterns. Take (Y,A) as an example. Let all categories of Y be arranged along the column axis, while all categories of *A* are arranged along the row axis. Then, the resultant contingency table, denoted as <Y,A>, is constructed as a rectangle array of cell counts. If we apply suitable permutations on the column and row axes, by aggregating unoccupied zero cells as much as possible, associative patterns and relations between Y and *A* become graphically visible. All information theoretic measurements used here are invariant with respect to row and column permutations. Further, even though the size of the contingency table can be very large, we still can visualize the global and large-scale pattern formations contained in <Y,A> if we tune the zooming.

The aforementioned associative patterns in fact can be numerically evaluated via various versions of conditional entropies (CE)s by basically treating <Y,A> as a 2D histogram of bivariate (Y,A). Given a column, say Y=y, we define a discrete conditional variable. Its Shannon entropy is calculated on this column’s vector of proportions, i.e., cell counts divided by its column sum, and is denoted as H[A|Y=y]. Across all columns, we calculate the weighted sum of H[A|Y=y] with respect to the weighting scheme of column-sum proportions. This is the overall or expected conditional entropy (CE) denoted as H[A|Y]. Likewise, along the row axis, each row of <Y,A>, say A=a, defines a conditional random variable with a CE denoted H[Y|A=a]. Then, we calculate the expected CE H[Y|A] as a properly weighted sum of the collection of row-wise CEs {H[Y|A=a]}. The column-wise and row-wise marginal entropies are denoted by H[Y] and H[A], respectively.

The intuitive meaning of H[A|Y] and H[Y|A] is visible through their contingency tables. This CE H[Y|A] conveys the amount of expected remaining uncertainty in Y after knowing *A* [13]. Conversely, by knowing Y, the CE H[A|Y] conveys the amount of remaining uncertainty in *A*. In other words, the two conditional entropy drops, i.e., the differences H[Y]−H[Y|A] and H[A]−H[A|Y] indicate the amount of information shared between *A* and Y (see also the review paper [18]):

$$\begin{array}{ccc} H[Y]-H[Y|A] & = & H[A]-H[A|Y]\\ & = & H[A]+H[Y]-H[A,Y]\\ & = & I[Y;A]. \end{array}$$

where H[A,Y] is the joint Shannon entropy of bivariate variable (Y,A), while I[Y;A] is the mutual information between Y and *A*. It is worth emphasizing the fact that I[Y;A] is the CE drop of *A* from the CE of Y.

Next, we consider the CE drop of bivariate (A,B) from the CE of Y. Their conditional mutual information given Y is calculated as:

I[A;B|Y]=H[A|Y]+H[B|Y]−H[(A,B)|Y].

Then, we can decompose the CE drop of (A,B) from the CE of Y into the following two key components: (1) the sum of the individual CE drops of *A* and *B* and (2) the difference in the conditional and marginal mutual information of *A* and *B*:

$$\begin{array}{ccc} H[Y]-H[Y|(A,B)] & = & H[(A,B)]-H[(A,B)|Y];\\ & = & H[A]+H[B]-I[A;B]-\{H[A|Y]+H[B|Y]-I[A;B|Y]\};\\ & = & \{H[Y]-H[Y|A]+H[Y]-H[Y|B]\}+\{I[A;B|Y]-I[A;B]\}. \end{array}$$

This simple difference {I[A;B|Y]−I[A;B]} indeed conveys the essence of interpretable meaning of conditional mutual information and plays a key role in our selection protocol for major factors. We first consider the case of *A* and *B* being marginally stochastically independent, in which case the marginal mutual information I[A;B]=0. Then, the positivity of I[A;B|Y] indicates that *A* and *B* obtain some degree of dependency under the constraint posed by the values of Y. The larger I[A;B|Y] is, the higher the degree of conditional dependency of *A* and *B* is. A high degree of conditional dependency of *A* and *B* given Y can occur via two scenarios: either the union A⋃B forms an essential constituent mechanism, or so-called major factor, within the dynamics of Y, or *A* and *B* indeed work as two separate mechanisms within the dynamics of Y. The former scenario requires a higher degree of conditional dependency than the latter one does.

The exact same interpretation is valid if the difference {I[A;B|Y]−I[A;B]} is significantly larger than zero even when the pair *A* and *B* are marginally stochastically dependent. This positivity of I[A;B|Y]−I[A;B] acts as the so-called “ecological effect”: the whole is larger than the sum of its parts. The implications of such ecological effects are particularly essential in this paper because we make use of the process of identifying a vital collection of major factors of Y as an avenue for understanding the mechanisms underlying Y.

On the other hand, this difference I[(A,B)|Y]−I[A;B] could be nearly zero or even negative when *A* and *B* are highly associated. For example, A=C⋃D and B=C⋃E, where {C,D,E} are mutually stochastically independent. For example, let Y=G(A,B) with G(.) defined by an “additive operation” acting on two separated “multiplicative operations” among member features of *A* and *B*, respectively. Then, we have the following:

$$\begin{array}{ccc} I[A;B] & = & H[C]\\ I[A;B|Y] & = & H[Y,A]+H[Y,B]-H[Y]-H[Y,A,B] \end{array}$$

The negative sign of I[(A,B)|Y]−I[A;B] is very likely if *C* plays an overwhelmingly more dominant role than *D* and *E* do in Y, since I[A;B|Y] is around zero. In this case, at most, either *A* or *B* is a candidate for the major factor of Y, but not both. This choice of major factor is a conservative means of decision-making.

However, as will be described in the subsection after the next one, it is realistic that Y=G(A,B,…) with G(.) being a rather complicated structural function of many other feature sets beyond *A* and *B*. When encountering a negative sign of I[(A,B)|Y]−I[A;B] and knowing *A* and *B* being rather distinct, we would still adopt the above conservative decision-making in determining whether either *A* or *B* is a potential candidate for the major factor. This decision-making is meant to avoid redundant complexity along the avenue of understanding the dynamics of Y. It is not equivalent to a declaration that both *A* and *B* cannot be a major factor simultaneously in theory.

With the above concepts of CE, CE drop and their relations with conditional mutual information, we next turn to a description of the criteria underlying our selection protocol. The task of understanding Y via $\left\{V_{1},\ldots ,V_{K}\right\}$ is defined by finding a collection of major factors of Y, denoted as $\left\{A_{m}^{*}|m=1,2,\ldots ,M\right\}$, such that each $A_{m}^{*}$ individually is “confirmable” and “irrepaceable” regarding information about Y. Let the cardinality of $A_{m}^{*}$ be denoted as $|A_{m}^{*}|$, so that $A_{m}^{*}$ is an order-$|A_{m}^{*}|$ major factor. Below, we define the two criteria, “confirmable” and ”irrepaceable”, for a feature set *A* to be a major factor of Y:

**[C1: confirmable]**: A feature set *A* is confirmable if a feature set $\tilde{A}$ is obtained by substituting any feature member of *A* with a feature that is completely independent of Y and *A*; we have I[Y;A], which is significantly larger than $I[Y;\tilde{A}]$.
**[C2: irrepaceable]**: A feature subset *A* is replaceable if $I\left[Y;A\right]\leq I\left[Y;A_{1}\right]+I\left[Y;A_{2}\right]$ for any compositions of *A*, i.e., $A=A_{1}⋃A_{2}$ and $A_{1}⋂A_{2}=\emptyset$. For *A* to be considered irreplaceable, we require that *A* is not replaceable and simultaneously satisfies the following two extra conditions: (a) its CE drop is larger than the sum of the top-ranked CE drop and at least |A|-times its complementary feature subset’s CE drop; (b) the candidate *A* joins with any already identified major factor $A_{m}^{*}$, and their CE must achieve $I\left[Y;A⋃A_{m}^{*}\right]\geq I\left[Y;A\right]+I\left[Y;A_{m}^{*}\right]$.

The criterion [C1: confirmable] is mainly used as a reliability check. It would be carried out via two algorithms developed later in this section. As for criterion [C2: irrepaceable], we elaborate as follows. The condition (a) ensures that some kind of structural dependency among all subsets of *A* is embraced under the constraints imposed by Y, not merely the occurrence of ecological effects. This condition indeed allows the following case to happen: $A_{m}^{*}\subset A_{m^{\prime }}^{*}$ for $\left(m,m^{\prime }\right)$ with $|A_{m}^{*}\left|<\right|A_{m^{\prime }}^{*}|$ and $A_{m^{\prime }}^{*}-A_{m}^{*}=B$ in the sense that *B* is the complementary of $A_{m}^{*}$ in $A_{m^{\prime }}^{*}$. This selection of $A_{m^{\prime }}^{*}$ is realistic only when the CE drop of $A_{m^{\prime }}^{*}$ minus the CE drop of $A_{m}^{*}$ is many times larger than *B*’s CE drop. In other words, $A_{m}^{*}$ and *B* have to form some “strong” bonds under the conditioning of Y in order to jointly become a higher-order major factor. In summary, these two criteria are designed to drastically reduce, or even avoid, the possibility of overestimating the significance of any candidate features and feature sets.

For the convenience of checking this condition, we routinely calculate and report the CE drop of *A* minus the top-ranked CE drop of its feature subsets and call this CE drop difference the “successive CE drop”. This quantity of “successive CE drop” is calculated and reported in tables under the name “SCE drop” for distinguishing with a CE drop referred to a feature set when developing our selection protocol throughout this paper.

The condition (b) again ensures the ecological effect among identified major factors. It is obvious but worth mentioning the difference between the conditions (a) and (b). Condition (a) sets a very high bar for building up any high-order major factors, such as order-3 or higher, while condition (b) simply requires the fulfilment of the ecological effect for any two major factors to coexist. In other words, there still exists potential for the union of two identified major factors to become a high-order major factor. However, the requirement via condition (a) is rather difficult to fulfil.

### 3.1. A Simple Illustrative Example for a Single System

In this subsection, we illustrate how to use the two criteria [C1: confirmable] and [C2:irrepaceable] for selecting major factors underlying a designated response variable Y. We begin with a rather simple example, with Y=Y being a 1D feature *Y* specified by the following analytic structure:

$$Y=V_{1}+sin\left(2\pi \left(V_{2}+V_{3}\right)\right)+\varepsilon ,$$

where the continuous response variable *Y* is defined by an additive operation acting on two mechanisms respectively specified by two major factors: $\left\{V_{1}\right\}$ and $\left\{V_{2},V_{3}\right\}$, and a normal noise $\varepsilon ∼\frac{1}{10}N\left(0,1\right)$ [14]. There are 10 covariate features, denoted by $\left\{V_{1},\ldots ,V_{10}\right\}$. They are mutually independent and identically distributed according to Uniform[0,1]. The simulated data set is an ensemble of one million $\left(10^{6}\right)$ of 11D vectors of $\left(Y,V_{1},\ldots ,V_{10}\right)$. After each variable has been categorized with respect to its own histogram with 10 uniform bins, we correspondingly retain their notations for expositional simplicity.

We illustrate the identifications of these two major effects and their ecological effect in terms of CEs and CE drops. For this goal, we first calculate the CEs for all possible $2^{10}\left(=1024\right)$ feature sets, on which our decision-making is based. However, due to the large size of the resulting file, we only report two tables of CEs and successive CE drops (SCEs): (1) Table 1, with the 10 top-ranked CEs; (2) Table 2, with the 10 top-ranked SCE drops of feature combinations across one-feature through four-feature settings. As a reminder, an SCE drop of a feature set is calculated as the amount of CE drop from the lowest CEs among all its possible feature subsets. Such an SCE drop is convenient for determining the amount of conditional mutual information when identifying potential candidates for major factors. The CE of *Y*, or the so-called 0-feature CE, is calculated as being equal to 2.0077. The observed CE-related patterns are listed below to motivate our selection protocol. A flowchart of this protocol will be given in the next subsection.

#### Selection of Major Factors in a Simple System

**Figure 1:**
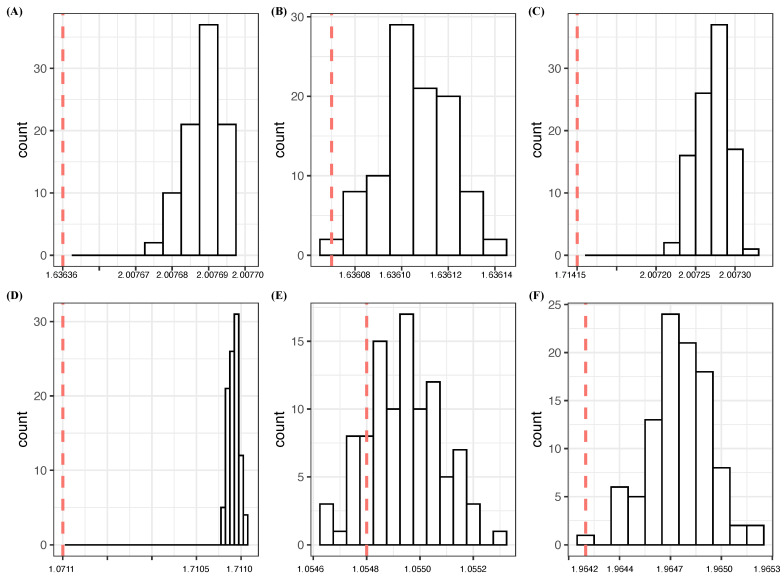
[C 1: confirmable] testings in the simple experiment: (**A**) $V_{1}^{*}$; (**B**) $V_{1}\_V_{9}^{*}$; (**C**) $V_{2}\_V_{3}^{*}$; (**D**) $V_{1}\_V_{2}\_V_{3}^{*}$; (**E**) $V_{1}\_V_{2}\_V_{3}\_V_{8}^{*}$; (**F**) $V_{5}\_V_{7}\_V_{8}^{*}\_V_{10}$. Each red dotted line indicates the conditional entropy given the feature set, and the histogram is the conditional entropies of samples generated with the *-marked feature being replaced by a random noise feature.

1 Based on the one-feature settings in Table 1 and Table 2, $V_{1}$ achieves the lowest CE of 1.6364 and the highest CE drop of 0.3703 (=2.0077−1.6364), while the rest of the features, $V_{2}$ through $V_{10}$, have basically zero CE drops. It is important to note that $V_{2}$ and $V_{3}$ are jointly, but not individually, involved in *Y*. These observations confirm that $V_{1}$ is a candidate for an order-1 major factor of *Y* with high potential. It satisfies the criterion [C1: confirmable], as seen in panel (A) of Figure 1, built based on Algorithm 1, which is given in the next subsection.
2 With regard to the two-feature setting, $V_{1}\_V_{9}$ achieve the lowest CE of 1.63607 with a nearly zero SCE drop from $V_{1}$. Hence, it is replaceable, and so are all the feature pairs: $V_{1}\_V_{k}$ with k=2,…,8,10. As seen in panel (B) of Figure 1, indeed, their CEs can be achieved by coupling $V_{1}$ with a random noise feature. Thus, they fail the test of criterion [C1: confirmable] for order-2 major factors. These observations indeed support $V_{1}$ as a candidate for an order-1 major factor of *Y*.
  In contrast, the feature pair $V_{2}\_V_{3}$ has the highest SCE drop from the minimum CEs of $V_{2}$ and $V_{3}$: 0.2935 (=2.0077−1.7142). This value is primarily due to the conditional mutual information, $I[V_{2},V_{3}|Y]$. A direct evaluation of $I[V_{2},V_{3})|Y]$ via $H(V_{2}|Y)+H(V_{3}|Y)-H(V_{2}V_{3}|Y)=2.3025+2.3025-4.3115=0.2935$, which is many times the CE drops of $V_{2}$ and $V_{3}$. Condition (a) of criterion [C2: irrepaceable] is fulfilled. Moreover, this feature pair passes the [C1: confirmable] test as, seen in panel (C) of Figure 1. These facts confirm $V_{2}\_V_{3}$ as a potential candidate for an order-2 major factor of *Y*.
3 In the three-feature setting, the triplet $V_{1}\_V_{2}\_V_{3}$ simultaneously achieves the lowest CE of 1.0711, and the highest SCE-drop: 0.5653. This triplet is confirmable, as seen in panel (D) of Figure 1. Further, analytic and numeric relationships among the CE drops of $V_{1}$, $V_{2}\_V_{3}$ and $V_{1}\_V_{2}\_V_{3}$ are given as follows:
  $$\begin{array}{ccc} & & \left\{H\left[Y\right]-H\left[Y|\left(V_{1},V_{2},V_{3}\right)\right]\right\}-\left\{H\left[Y\right]-H\left[Y|V_{1}\right]\right\}-\left\{H\left[Y\right]-H\left[Y|\left(V_{2},V_{3}\right)\right]\right\}\\ & = & I\left[V_{1};\left(V_{2},V_{3}\right)|Y\right]-I\left[V_{1};\left(V_{2},V_{3}\right)\right];\\ & = & I[V_{1};\left(V_{2},V_{3}\right)|Y]=0.2848. \end{array}$$
  where the marginal mutual information $I[V_{1};\left(V_{2},V_{3}\right)]$ of two mutually independent variables, $V_{1}$ and $\left(V_{2},V_{3}\right)$, is zero. Therefore, the extra CE drop is exactly equal to the conditional mutual information $I[V_{1};\left(V_{2},V_{3}\right)|Y]$. Thus, condition (b) of criterion [C2: irreplaceable] is fulfilled. In other words, they can simultaneously be declared as two identified major factors: $V_{1}$ and $\left(V_{2},V_{3}\right)$. Moreover, we also conclude that, with respect to condition (a), the triplet $V_{1}\_V_{2}\_V_{3}$ is not an order-3 major factor because its conditional mutual information $I[V_{1};\left(V_{2},V_{3}\right)|Y]$ is far from being “many times” the minimum CE drops of $V_{1}$ and $V_{2}\_V_{3}$.
4 Considering the four-feature settings, the quartets constructed by coupling $V_{1}\_V_{2}\_V_{3}$ with $V_{k}$ with k=4,…,10 achieve the lowest CEs, with rather uniform SCE drops of around 0.0162. Their CEs can be achieved by coupling $V_{1}\_V_{2}\_V_{3}$ with a random noise feature; see the quartet $V_{1}\_V_{2}\_V_{3}\_V_{8}$ in panel (E) of Figure 1. This fact indicates that none of $V_{k}$ with k=4,…,10 can couple with triplet $\left\{V_{1}\_V_{2}\_V_{3}\right\}$ to produce detectable effects on *Y*.
  On the other hand, the largest SCE drop of 0.0387 is achieved by a quartet of knowingly random features: $V_{5}\_V_{7}\_V_{8}\_V_{10}$. In fact, any quartets of $\left\{V_{k}|k=4,\ldots ,10\right\}$ achieve nearly the same CE drops. By using Algorithm 1, we show that the CEs of all these quartets can be achieved by replacing any one of the four features with a random noise feature. Therefore, they are definitely not potential major factors. Such CE drops are clearly due to the finite sample phenomenon.
5 Considering the five-feature through nine-feature settings, considerations similar to those of the four-feature setting hold. All CE drops are confirmed as being attributed to randomness and small row-sums.

Based on the results from the above five feature settings, we declare a collection of major factors, $\left\{V_{1},\left(V_{2},V_{3}\right)\right\}$, underlying the dynamics of *Y*. It is necessary to note that one order-1 and one order-2 major factors are declared simultaneously, not individually, because of condition (b) of criterion [C2: irreplaceable]. After declaring a collection of major factors, the understanding of Y=Y should be carried out through contingency tables of individual major factors, as well as their union against the *Y*.

It is worth emphasizing here that the essential check via Algorithm 1, as will be given in the next subsection, safeguards the reliability of all information theoretic measures used in our selection protocol against the finite sample phenomenon in the contingency table construction, which acts as the “curse of dimensionality” in distribution estimation. As a final remark, we also report that popular statistical feature selection approaches, such as AIC, BIC and MDL, which rely on structural and distributional assumptions, such as linearity, all fail even in this simple experiment.

### 3.2. Structural Formation and Major Factors in Complex Systems

In this subsection, we postulate a generic structural formation for settings of one single system as well as for a collection of systems. From each single complex system, a data point is measured and collected in a L+KD vector format. Let the first *L* components be measurements or categories of the response features denoted as $Y={\left(Y_{1},\ldots ,Y_{L}\right)}^{\prime }$, and the rest of the *K* components are measurements or categories of *K* one-dimensional covariate features denoted as $\left\{V_{1},\ldots ,V_{K}\right\}$. It is essential to note that one covariate feature, say $V_{K}$, is the categorical feature of system labels or IDs.

An unspecified complex structural relation between Y and $\left\{V_{1},\cdots ,V_{K}\right\}$ consists of a collection of *M* unknown constituent mechanisms, $\left\{F_{m}\left\{A_{m}^{*}\right\}|m=1,\ldots ,M\right\}$, in the following fashion:

(1) $$\begin{array}{cc} Y & =\left[\begin{array}{c} Y_{1}\\ Y_{2}\\ \vdots \\ Y_{L} \end{array}\right]≅G\left(F_{1}\left\{A_{1}^{*}\right\},F_{2}\left\{A_{2}^{*}\right\},\ldots F_{M}\left\{A_{M}^{*}\right\}\right)+\varepsilon . \end{array}$$

Here, we do not have any a priori knowledge or assumptions of *M*, functional forms of $F_{m}\left\{\cdot \right\}$, the random noise ε or the governing structural function G(·). We only focus on identifying the feature memberships of each $A_{m}^{*}\left(\subset \left\{V_{1},\ldots .,V_{K}\right\}\right)$ with m=1,…,M. By acquiring these memberships, the layouts or patterns of constituent mechanisms within the dynamics underlying Y are supposed to be visible and explainable through the contingency tables of $A_{m}^{*}-vs-Y$ and ${⋃}_{m\in S}A_{m}^{*}-vs-Y$.

Therefore, our computational task can be simply described as discovering the collection of major factors $\left\{A_{m}^{*}|m=1,\ldots ,M\right\}$. This computational task primarily relies on information theoretic measures, reviewed in the previous subsection.

Next, we develop an algorithm, Algorithm 1, as a tool for reliability checking against the finite sample phenomenon and for the testing of criterion [C1: confirmable]. By performing both checking and testing upon a candidate feature set via Algorithm 1, we simulate the distribution of conditional entropies by substituting a member of this feature set by a random noise feature that is supposed to be stochastically independent of all response and covariate features. The idea of this algorithm is given as follows. Let a candidate feature set be $A=B⋃\{V_{k}\}$ with the 1D covariate feature $V_{k}$ having *h* categories. To substitute $V_{k}$ by a random noise feature, say ξ, we expand each row of the contingency table of B−vs−Y into χ rows. This expansion is done by redistributing each cell count of any targeted row via multinomial distribution with equal probabilities 1/χ. Then, the Shannon entropy is calculated upon each of χ newly created rows.

By performing such entropy calculations in the above row-by-row fashion, the conditional entropy is calculated for $A^{\prime }=B⋃\left\{\xi \right\}$. We repeat this process many times and then construct a histogram of conditional entropies, such as the histograms reported in Figure 1. For instance, by applying Algorithm 1, it is clear that $V_{1}$ and $\left(V_{2},V_{3}\right)$ are major effects, while $\left(V_{1},V_{9}\right)$ and $\left(V_{1},V_{2},V_{3},V_{8}\right)$ are not, as in the previous subsection.
**Algorithm 1** Simulate a contingency table with adding a random noise feature.
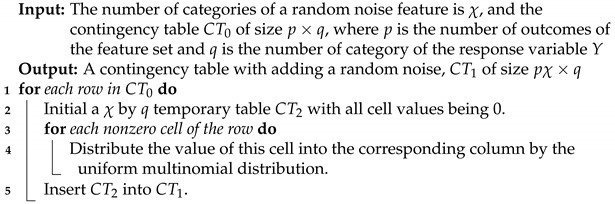


**Figure 2:**
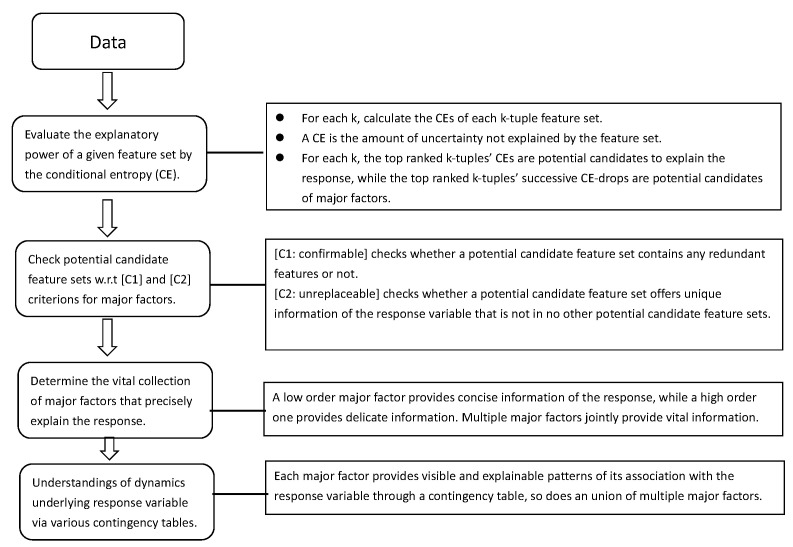
Flowchart of CEDA-based selection protocol for major factors.

At the end of this subsection, we present a flowchart (Figure 2) of our selection protocol for major factors. This protocol will be used throughout the entire paper.

### 3.3. A Complex Illustrative Example for One System

In this subsection, we consider another illustrative example with only eight covariate features, but having a much more complex analytic structure than the previously discussed simple example. Again, the response variable Y=Y is still single-dimensional, i.e., L=1. The goal of this illustrative example is to further demonstrate our feature selection protocol via the use of CE and CE drops when discovering high-order major factors. The complex analytic structure of *Y* is given as follows:

$$Y=V_{2}+2V_{1}V_{7}+sin\left(2\pi \left(V_{3}+V_{6}\right)\right)+6V_{1}V_{3}V_{6}+sin\left(2\pi \left(V_{1}+V_{6}+V_{7}+V_{8}\right)\right)+\varepsilon ,$$

where $V_{1}$ through $V_{8}$ are mutually independently Uniform[0,1] distributed, and a normal noise $\varepsilon ∼\frac{1}{10}N\left(0,1\right)$. The $V_{4}$ and $V_{5}$ play the role of pure random noises with respect to *Y*. With Algorithm 1, there is no need to know this fact in our computational protocol.

There are 5(=M) major factors of *Y*. They are: (1) $\left\{V_{2}\right\}\left(=A_{1}^{*}\right)$; (2) $\left\{V_{1},V_{7}\right\}\left(=A_{2}^{*}\right)$; (3) $\left\{V_{3},V_{6}\right\}\left(=A_{3}^{*}\right)$; (4) $\left\{V_{1},V_{3},V_{6}\right\}\left(=A_{4}^{*}\right)$ and (5) $\left\{V_{1},V_{6},V_{7},V_{8}\right\}\left(=A_{5}^{*}\right)$. The members of major factors $A_{2}^{*}$ and $A_{3}^{*}$ overlap with major factors $A_{4}^{*}$ and $A_{5}^{*}$. Such overlapping characteristics create the intended complexity contained in this example.

Again, we simulate one million 9D continuous data vectors. The one million values of *Y* are then categorized into 18 categories indexed as 1 through 18. In this experimental example, we compute conditional entropies (CE) for all possible combinations of eight features and their SCE drops. In Table 3 and Table 4, we respectively report the eight top-ranked CEs and SCE drops across one-feature to five-feature settings. The CE of *Y* is 1.9945 as the 0-feature CE. All computed patterns via our selection protocol are based on all possible feature combinations and summarized below in a step-by-step fashion:

#### Selection for Major Factors in a Complex System

1 Based on the one-feature setting in Table 3 and Table 4, the one-feature CEs of $V_{4}$ and $V_{5}$, as expected, are very close to the CE of *Y*. In contrast, the four features $V_{1}$, $V_{2}$, $V_{3}$ and $V_{6}$ have sizeable CE drops, so they are tentatively taken as potential candidates for order-1 major factors and await further confirmation. The remaining two features, $V_{7}$ and $V_{8}$, seem less likely to be order-1 major factors. Though their CE drops are small, there are many folds of CEs of $V_{4}$ and $V_{5}$. All individual CE drops interestingly reflect their degrees of involvement in *Y*.
2 In the two-feature setting, both feature pairs, $\left(V_{3},V_{6}\right)$ and $\left(V_{1},V_{7}\right)$, pass their tests for criterion [C1: confirmable] when applying Algorithm 1. The feature pair $\left(V_{3},V_{6}\right)$ achieves the lowest CE of 1.8543, which is far below the range of the simulated CE distribution of $\left(V_{3},\xi \right)$, with mean 1.9666 and sd 2.534020 × 10${}^{-5}$. The CE of feature pair $\left(V_{1},V_{7}\right)$ is ranked third, with a value of 1.8812, which is also far below the range of the simulated CE distribution of $\left(V_{1},\xi \right)$, with mean 1.9400 and sd 2.4143 × 10${}^{-5}$. The feature pair $\left(V_{3},V_{6}\right)$ also achieves the largest CE drop, 0.1402(=1.9945−1.8543), which is much larger than the sum of their individual CE drops, 0.0652(=0.0327+0.0325). This fact implies that the feature pair $V_{3}\_V_{6}$ is likely an order-2 major factor due to their large conditional mutual information $I[\left(V_{3},V_{6}\right)|Y]$. In order to further satisfy the compositional components of the criterion [C2: irreplaceable], we do not declare either $V_{3}$ or $V_{6}$ as order-1 major factors. Via a similar argument, $\left(V_{1},V_{7}\right)$ is also a potential candidate for an order-2 major factor when both $V_{1}$ and $V_{7}$ are declared not order-1 major factors.
  In contrast, all the other feature pairs are not major factors primarily because they fail to satisfy criterion [C2: irreplaceable]. For example, $\left(V_{1},V_{2}\right)$ achieves the second-highest CE with an SCE drop of 0.1268, which turns out to be very close to the sum of their individual CE drops: 0.1188. Therefore, the feature pair $\left(V_{1},V_{2}\right)$ is not a major factor. Based on this line of reasoning, our feature selection protocol suggests that $\left(V_{3},V_{6}\right)$ and $\left(V_{1},V_{7}\right)$ are the only potential candidates for order-2 major factors.
3 With regard to the three-feature setting, using Algorithm 1, the triplet feature $\left(V_{1},V_{3},V_{6}\right)$ passes the test of criterion [C1: confirmable] by having a CE of 1.6042, which is far beyond the range of the simulated CE distribution of $\left(V_{1},V_{3},\xi \right)$ with mean 1.9007 and sd 6.825321 × 10${}^{-5}$. It achieves an SCE drop of 0.3903. Thus, its CE drop is much larger than the sum of the CE drops of pairs $\left(V_{3},V_{6}\right)$ and $V_{1}$, 0.1941=0.1402+0.0539. In other words, their conditional mutual information $I\left[\left\{V_{1}\right\},\left\{V_{3}\_V_{6}\right\}|Y\right]=0.3354\left(=0.3903-0.0539\right)$ is many times the individual CE drop of $V_{1}$: 0.0539. Condition (a) of criterion [C2: irreplaceable] is fulfilled.
  In comparison, it is also essential to observe that the SCE drop of $\left(V_{2},V_{3},V_{6}\right)$ is only 0.0903, so the conditional mutual information $I[\left\{V_{2}\right\},\left\{\left(V_{3},V_{6}\right)\right\}|Y]$ is calculated as 0.0324(=0.0903−0.0579), which is approximately half of the CE drop of $V_{2}$ and one quarter of the CE drop of $\left(V_{3},V_{6}\right)$. These observations together strongly support that $\left(V_{1},V_{3},V_{6}\right)$ is the only potential candidate for an order-3 major factor, even if it is overlapping with major factor $\left(V_{3},V_{6}\right)$. These reasons once again indicate that $V_{1}$ should not be an order-1 major factor.
4 In the four-feature setting, Algorithm 1, $\left(V_{1},V_{3},V_{6},V_{7}\right)$ passes the test of [C1: confirmable] by having a CE of 1.4262, being far beyond the simulated CE distribution of $\left(V_{1},V_{3},V_{6},\xi \right)$ with mean 1.5748 and sd 1.7478 × 10${}^{-4}$. However, its CE drop is indeed very close to the sum of the CE drops of $\left(V_{1},V_{3},V_{6}\right)$ and $V_{1}\_V_{7}$. Likewise, the CE drop of $\left(V_{1},V_{2},V_{3},V_{6}\right)$ is smaller than the sum of the CE drops of the major factors: $\left(V_{1},V_{3},V_{6}\right)$, $\left(V_{3},V_{6}\right)$ and $V_{2}$. Due to their overlapping in feature members, we can conclude that their corresponding values of conditional mutual information are nearly equal to their marginal values of mutual information. In other words, these quartets do not achieve the ecological effects when conditioning on *Y*. Therefore, they are not order-4 major factors. This fact is coherent with criterion [C2: irreplaceable].
  As for the feature quartet $\left(V_{1},V_{6},V_{7},V_{8}\right)$, it is a different case. This feature quartet passes the test criterion [C1: confirmable] since, via Algorithm 1, its CE of 1.7012 is far below the range of the simulated CE distribution of $\left(V_{1},V_{6},V_{7},\xi \right)$ with mean 1.785142 and sd. 1.937145 × 10${}^{-4}$. Although it achieves a CE value that is not ranked among the top eight, its SCE drop for $\left(V_{1},V_{7}\right)$ is 0.1800, which is more than three times the individual CE drop of $V_{1}$, six times the CE drop of $V_{6}$ and many times the CE drop of $V_{8}$. Further, the inclusion of $V_{8}$ indicates that $\left(V_{1},V_{6},V_{7},V_{8}\right)$ satisfies the criterion [C2: irreplaceable]. Thus, this quartet is a potential candidate for a order-4 major factor. The rest of the feature quartets in both tables are not order-4 major factors.
5 It is noted that $V_{2}$ does not appear in any of the four identified potential candidates for major factors of various orders, $\left(V_{3},V_{6}\right)$, $\left(V_{1},V_{7}\right)$, $\left(V_{1},V_{3},V_{6}\right)$ and $\left(V_{1},V_{6},V_{7},V_{8}\right)$, and has a larger individual CE drop than $V_{1}$. Thus, $V_{2}$ is likely an order-1 major factor, but not $V_{1}$, $V_{3}$ or $V_{6}$, from the perspective of criterion [C2: irreplaceable].
6 Across five-feature to eight-feature settings, except for $\left(V_{1},V_{2},V_{3},V_{6},V_{7},V_{8}\right)$, none of the subsets of variables passes the the test of [C1: confirmable] criterion when applying Algorithm 1. For instance, the feature set $\left(V_{1},V_{3},V_{6},V_{7},V_{4}\right)$ achieves the lowest CE, 1.2152, in the five-feature setting. This CE falls within one sd of the mean, 1.2157, of the simulated CE distribution of $\left(V_{1},V_{3},V_{6},V_{7},\xi \right)$, with sd 4.4982 × 10${}^{-4}$. As for the feature set $\left(V_{1},V_{2},V_{3},V_{6},V_{7},V_{8}\right)$, it fails criterion [C2: irreplaceable] because it is a union of all the already identified major factors.

Here, we conclude that [C1: confirmable], aided by Algorithm 1, and criterion [C2: irreplaceable] work well as the backbones of our feature selection protocol for identifying the collection of major factors even in this example of complex dynamics.

## 4. Gerrit Cole’s Pitching Dynamics

In this section, we apply the selection protocol developed and illustrated in the previous sections on Gerrit Cole’s slider and fastball pitching dynamics over three consecutive MLB seasons: 2017, 2018 and 2019. Two structured data sets contain 2042 slider pitches and 5328 fastball pitches obtained from PITCHf/x and Statcast, respectively. Upon each pitching dynamics, we successively study two settings separately with 2D response variables $Y=(pfx_{X},pfx_{Z})$ and Y=(aX,aZ). We denote a collection of biomechanical and physical covariate features as $\left\{V_{1},\ldots ,V_{K-1}\right\}$, with K=12 for the former setting and K=10 for the latter setting. We commonly use the *K*-th feature $V_{K}=pitN$ to represent the three categorical pitcher seasons. Within each of these two pitching dynamics, we explore and evaluate the potential effects of heterogeneity across the three seasons as an appropriate means of addressing the question raised at the beginning of the Introduction.

### 4.1. Gerrit Cole’s Slider Pitching Dynamics

**Figure 3:**
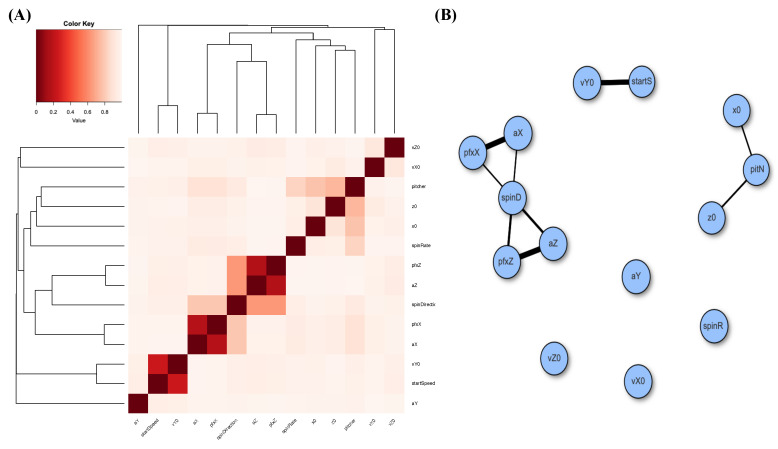
Associative patterns among 14 features of Gerrit Cole’s slider: (**A**) heatmap based on MCE matrix; (**B**) network built with linkages having thickness proportional to one minus pairwise MCE and subject to a threshold of 0.2 (=1.0−MCE).

At first, we evaluate the pairwise MCE associations among all 14 features: response and covariate. As mentioned in the Introduction, this association of two categorized or categorical features is evaluated on their contingency table. The heatmap based on the MCE matrix of Gerrit Cole’s slider pitches and its corresponding network are reported in the two panels of Figure 3. It is noted that the associative patterns among the 14 features pertaining to Gerrit Cole’s slider pitches appear simple. We see several disconnected small communities and isolated nodes. Such disconnecting patterns indicate that, overall, fewer associations are found among these 14 features. In particular, the spinR is disconnected from pitN with respect to a threshold. These disconnecting patterns would exert effects on our selection for major factors.

We first consider the slider case with the 2D response variable $Y=(pfx_{X},pfx_{Z})$. These two directional movements together with vY0 or startSp critically contribute to this pitcher’s capability of successfully dealing with batters. Our selection results via CEs are acquired based on all possible combinations of 12 covariate features. In Table 5, we report the feature sets achieving the top 12 CEs across one-feature to four-feature settings. We summarize and itemize our findings attached with reasoning as follows. The entropy of Y or 0-feature entropy is calculated as 4.6623.

**Figure 4:**
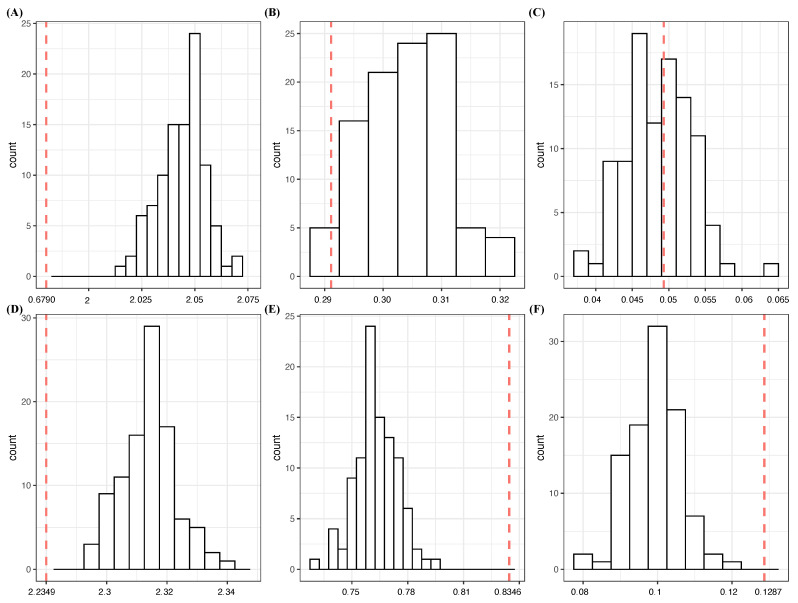
Determine when to stop CEDA-based selection for major factors: (**A**) (aZ,aX); (**B**) $\left(aX,aZ,vY0^{*}\right)$; (**C**) $\left(aX,aZ,aY,x0^{*}\right)$ on $Y=(pfx_{X},pfx_{Z})$; (**D**) $\left(spinD,vX0^{*}\right)$; (**E**) $\left(vZ0,spinR,aY^{*}\right)$; (**F**) $\left(x0,vZ0,spinR,aY^{*}\right)$ on Y=(aX,aZ). Each red dotted line indicates the conditional entropy given the feature set, and the histogram is the conditional entropies of samples generated with the *-marked feature being replaced by a random noise feature.

1 In the one-feature setting, the aX, aZ and spinD achieve the three top-ranked CEs. Thus, they are natural candidates for order-1 major factors. It is noted that pitN is ranked the lowest, while spinR is ranked the fourth.
2 With regard to the two-feature setting, the three top-ranked pairs, (aX,aZ), (aX,spinD) and (aZ,spinD), achieve low CE values with CE drops that are all less than the sum of their individual drops. Even though these pairs pass the test for criterion [C1: confirmable]—see panel (A) of Figure 4—they fail on criterion [C2: irreplaceable]. In other words, only one of them could be an order-1 major factor. This conclusion is further confirmed by the CE of triplet (aX,aZ,spinD), 0.5539, which only achieves a very small SCE drop from (aX,aZ), proving insignificant in comparison with the top eight triplets of the three-feature setting in Table 5.
  The rest of the nine feature pairs are not order-2 major factors. Three collections of two separate order-1 major factors, {aZ,x0}, {aZ,spinR} and {aZ,z0}, are selected, since all candidate triplet collections fail either the test for criterion [C1: confirmable] or the criterion [C2: irreplaceable] in the three-feature setting below. Further, these three pairs achieve the lowest CEs and satisfy condition (b) of the criterion [C2: irreplaceable].
  In sharp contrast, we found that pairs of {aY,vZ0,vX0,vY0,spinR,x0,z0}, excluding (vX0,vZ0) and (x0,z0), achieve successive SCE drops that are larger than three times another member’s individual CE drops. Thus, these pairs satisfy the criterion [C2: irreplaceable]. They all satisfy the test for criterion [C1: confirmable]. However, these pairs’ candidacy of order-2 major factors would fail if aZ is the choice for the order-1 major factor because of criterion [C2: irreplaceable]. It is also noted that none of the pairs including {pitN}×{aY,vZ0,vX0,vY0,spinR,x0,z0} satisfy the criterion [C2: irreplaceable]. This is a striking manifestation of homogeneity within the dynamics of $Y=(pfx_{X},pfx_{Z})$ across the three seasons.
3 In the three-feature setting, the 10 top-ranked triplets all contain (aX,aZ). For instance, the triplet (vY0,aX,aZ) achieves a CE drop from the SCE of (aX,aZ), which is less than the individual CE drop of vY0: 0.4670. Moreover, note that the CE of (vY0,aX,aZ) is somehow achievable by triplets (aX,aZ,ε); see panel (B) of Figure 4. In other words, both criteria [C1: confirmable] and [C2: irreplaceable] fail, and so do the rest of the nine triplets.
  As for the 11th and 12th ranked triplet collections, {aZ,x0,aY} and {aZ,z0,aY}, they fail both criteria as well. Even though the two pairs (x0,aY) and (z0,aY) are candidates for order-2 major factors, the choice of aZ as an order-1 major factor fails both.
  On the other hand, all the feature triplets of {aY,vZ0,vX0,vY0,spinR,x0,z0} achieve successive CE drops of at least 1.6000. These seemingly large successive CE drops indeed satisfy the criterion [C2: irreplaceable]. However, they all fail the test for [C1: confirmable]. Thus, these high CE drops are primarily due to the finite sample phenomenon.
4 Considering the four-feature setting, the CE of quartet (x0,aX,aY,aZ), which achieves the lowest CE, is comparable with the CEs of (x0,aX,aZ,ε). Based on panel (C) of Figure 4, we are certain that the major factor selection computations on the response $Y=(pfx_{X},pfx_{Z})$ should not go beyond the four-feature setting.

Based on the above computations, we identify three collections, {aZ,x0}, {aZ,spinR} and {aZ,z0}, of two order-1 major factors for $Y=(pfx_{X},pfx_{Z})$ of Gerrit Cole’ slider. aZ plays a dominant role and bears significant effects on $Y=(pfx_{X},pfx_{Z})$. In contrast, spinR plays only an alternative minor role. Moreover, the homogeneity is evidently seen through the abundance and diversity of candidates for order-2 major factors with detachment with pitN. To further confirm the presence of homogeneity, we investigate slider pitching dynamics with Y=(aX,aZ).

With response variable Y=(aX,aZ), we acquire the major factor selection results based on the CEs of all the possible feature sets of 10 covariate features, but we only report the top 10 feature sets across one-feature to four-feature settings in Table 6 for Gerrit Cole’s slider. We summarize and itemize our findings attached with reasoning as follows. The entropy of Y or 0-feature entropy is calculated as 4.6652.

1 In the one-feature setting, the spinD achieves the largest CE drop, 1.5694, while the remaining features only achieve individual CE drops of less than 0.5000. Therefore, spinD is an apparent candidate for an order-1 major factor of Y=(aX,aZ). It is also noted that the CE and CE drop of pitN have the lowest rank in this one-feature setting. This is a significant sign of the homogeneity in Gerrit Cole’s slider pitching dynamics across the three considered seasons.
2 Regarding the two-feature setting, the top eight pairs are all related to spinD and the 9th and 10th pairs are also related to spinR, but not spinD. Among the eight pairs, we select three collections, {spinD,vZ0}, {spinD,vZ0} and {spinD,z0)}, as the collections of two order-1 major factors of Y=(aX,aZ). In fact, they satisfy the two criteria.
  The top eight pairs achieve only slightly lower CE as compared to the 9th and 10th pairs, while these two pairs indeed achieve very significant CE drops that are larger than the sum of the two members’ individual CE drops. In fact, pairs of {aY,vZ0,vX0,vY0,spinR,x0,z0}, excluding (vX0,vZ0) and (x0,z0), are candidates for order-2 major factors by achieving significant CE drops that satisfy the two criteria, [C1: confirmable] (see panel (D) of Figure 4) and [C2:irreplaceable]. This fact means that these six biomechanical features together with spinR are tightly bonded together in constituting the dynamics of Y=(aX,aZ), not merely the spinR. The pitN does not involve any candidates for order-2 major factors at all.
3 All triplets of {aY,vZ0,vX0,vY0,spinR,x0,z0} achieve significant CE drops and three of them are in the top 10 list. However, they all neither satisfy the criterion [C1: confirmable], based on panel (E) of Figure 4, nor the criterion [C2: irreplaceable]. Therefore, we can conclude that there are no order-3 major factors.
4 Indeed, based on panels (E) and (F) of Figure 4, we are confident that our selection should stop at the three-feature setting.

Based on the above findings, spinD as an order-1 major factor definitely contributes significantly to Y=(aX,aZ). The alternative choices, vX0, vZ0 and z0, help spinD contribute slightly more toward Y as an alternative order-1 major factor. Regarding the response Y=(ax,az), the three panels (D-F) of Figure 4 strongly indicate that we should stop our major factor selection computations at the two-feature setting. This fact is primarily due to the finite sample phenomenon.

As for spinR, it surely has its roles in $Y=(pfx_{X},pfx_{Z})$ and Y=(aX,aZ). However, its importance is only comparable with any biomechanical one of {aY,vZ0,vX0,vY0,x0,z0}. Therefore, a fair and naturally true statement is that the increase in spinR observed in Gerrit Cole’s slider pitches over the three seasons is likely transformed into other factors, such as better control of $\left(pfx_{X},pfx_{Z}\right)$ in delivering slider pitches.

### 4.2. Gerrit Cole’s Fastball Pitching Dynamics

**Figure 5:**
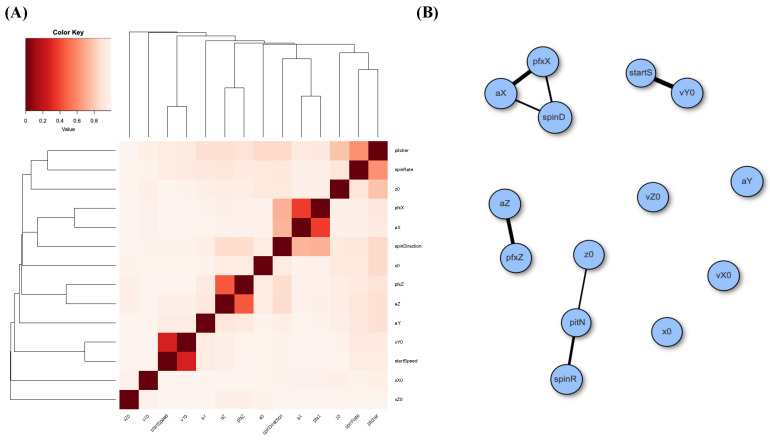
Associative patterns among 14 features of Gerrit Cole’s fastball: (**A**) heatmap based on MCE matrix; (**B**) network built with linkages having thickness proportional to one minus pairwise MCE and subject to a threshold of 0.2 (=1.0 −MCE).

Next, we also perform MCE computations for Gerrit Cole’s fastball over the three considered seasons. We build a heatmap for the 14 features and likewise construct a network via the same thresholding scheme; see panels (A) and (B) of Figure 5. The degree of disconnection among these feature nodes based on his fastball pitches is even more so than that of his slider case. In other words, many nearly stochastic independencies are observed here. Such independence would bear effects on the conditional mutual information argument when checking the criterion [C2: irreplaceable].

Again, we perform our selection computations for all possible feature sets among the 12 covariate features. Only the 12 top-ranked feature sets on CEs are reported in Table 7. We summarize and itemize our selection results regarding the dynamics of $Y=(pfx_{X},pfx_{Z})$. The entropy of Y is calculated to be 4.6710.

**Figure 6:**
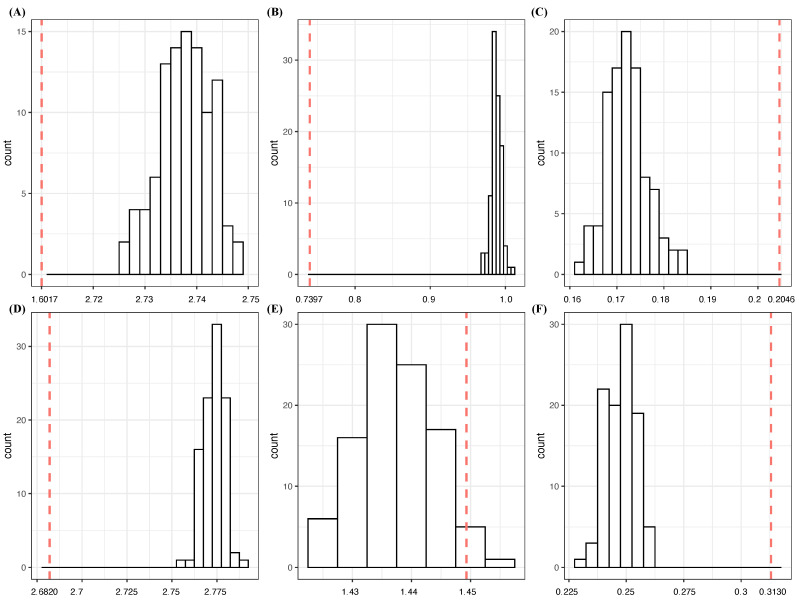
Determine when to stop CEDA-based selection for major factors: (**A**) $\left(aX,aZ^{*}\right)$; (**B**) $\left(aX,aZ,vY0^{*}\right)$; (**C**) $\left(aX,aZ,vX0,vY0^{*}\right)$ on $Y=(pfx_{X},pfx_{Z})$; (**D**) $\left(spinD,aY^{*}\right)$; (**E**) $\left(spinD,vZ0,aY^{*}\right)$; (**F**) $\left(startSp,x0,vZ0,vX0^{*}\right)$ on Y=(ax,az). Each red dotted line indicates the conditional entropy given the feature set, and the histogram is the conditional entropies of samples generated with the *-marked feature being replaced by a random noise feature.

1 With regard to the one-feature setting, the three features spinD, aX and aZ achieve the three lowest CEs, with CE drops being more than 1.3000. These three are candidates for order-1 major factors.
2 In the two-feature setting, again, the pair (aX,aZ) achieves the lowest CE with a CE drop of 3.0693, which is larger than the sum of their individual CE drops: 2.7730. Thus, this pair fails condition (a) but satisfies condition (b) of [C2: irreplaceable] by viewing it as a composition of two order-1 major factors: {aX,aZ}. In contrast, the two pairs, (aX,spinD) and (aZ,spinD), have CE drops smaller than the sum of their individual CE drops.
  The two feature pairs (vY0,aX) and (vY0,aZ) achieve SCE drops that are more than three times the CE drop of vY0. In other words, criterion [C2: irreplaceable] is satisfied for both pairs. They also pass the test for the criterion [C1: confirmable]. Thus, (vY0,aX) and (vY0,aZ) are candidates for order-2 major factors. This involvement of vY0 in these two pairs reveals the key aspect of fastball pitching dynamics, being distinct from the slider pitching dynamics of this pitcher.
  Again, all pairs of {aY,vZ0,vX0,vY0,x0,z0} and (vX0,spinR), (vY0,spinR) and (vZ0,spinR) are declared as candidates of order-2 major factors since they achieve significant SCE drops that are more than three times their individual CE drops. They also all satisfy the two criteria [C1: confirmable] (see panel (A) of Figure 6) and [C2: irreplaceable].
3 Upon the three-feature setting, as in the slider case reported in Table 5, the top-ranked three-feature triplet, (vY0,aX,aZ), satisfies the criterion [C1: confirmable]; see panel (B) of Figure 6. It fails condition (a), whereas it satisfies condition (b) of [C2: irreplaceable], since it can be seen as a composition of one order-1 and one order-2 major factors: {aX,(vY0,aZ)}. Thus, the triplet (vY0,aX,aZ) is not an order-3 major factor, but {aX,(vY0,aZ)} is a collection of major factors of dynamics of $Y=(pfx_{X},pfx_{Z})$. This very interesting result is not seen in the slider case. Note that the collections ranked third and fourth, namely {aX,(vZ0,aZ)} and {aX,(vX0,aZ)}, fail condition (b) of [C2: irreplaceable]. As for all triplets of {aY,vZ0,vX0,vY0,spinR,x0,z0}, they are not order-3 major factors, since they all fail criterion [C1: confirmable], even if they have rather large SCE drops.
4 According to panel (C) of Figure 6, our major factor selection endeavors should stop at the three-feature setting.

Similarly to the slider case, we investigate whether spinD and spinR bear major effects on Y=(aX,aZ). We perform our major factor selection computations for all possible feature sets of 10 features. Only the top 10 feature set on CEs are reported in Table 8. We summarize and itemize our findings across one-feature to four-feature settings. The zero feature’s entropy is 4.6913.

1 In the one-feature setting, the spinD achieves the largest CE drop that is more than the second-highest CE drop of spinR, 0.4233, by 1.0000. Thus, spinD is a natural candidate for an order-1 major factor of Y=(aX,aZ).
2 Upon the two-feature setting, the top nine pairs are all involved with spinD and only the 10th pair, (vZ0,spinR), does not involve spinD. These top nine pairs achieve much smaller CE drops than that of the 10th pair. In fact, the three pairs, (vX0,spinR), (vY0,spinR) and (vZ0,spinR), together with all pairs from the feature set {aY,vZ0,vX0,vY0,x0,z0}, are candidates for order-2 major factors. They achieve significant CE drops and satisfy the two criteria: [C1: confirmable] (see panel (D) of Figure 6) and [C2: irreplaceable].
3 All triplets in the top 10 list and triplets from {aY,vZ0,vX0,vY0,spinR,x0,z0} fail to satisfy the criterion: [C1: confirmable], based on panel (E) of Figure 6. There are neither order-3 major factors nor triplet collections of major factors.
4 Similarly, based on panels (E) and (F) of Figure 6, our major factor selection should stop at the three-feature setting.

### 4.3. Summary of Gerrit Cole’s Slider and Fastball Pitching Dynamics

We summarize all of our findings on Gerrit Cole’s slider and fastball across three MLB seasons in Table 9. Based on the findings listed in this table, we are confident that the Magnus effect through spinD has significant effects on Y=(aX,aZ), and consequently, it has major impacts on $Y=(pfx_{X},pfx_{Z})$. In other words, the major role of spinD in Gerrit Cole’s slider and fastball pitching dynamics is evident.

As for the roles of spinR in both pitching dynamics of Gerrit Cole, there exists an evident difference. In his slider case, spinR couples with aZ in the setting of $Y=(pfx_{X},pfx_{Z})$ as an alternative order-1 major factor having relatively minor effects. Meanwhile, in the fastball case, spinR couples with spinD and serves as an alternative order-1 major factor having minor effects on the dynamics of Y=(aX,aZ). These two pieces of evidence clearly reveal and reflect the minor role that spinR plays in Gerrit Cole’s slider and fastball pitching dynamics. Furthermore, it is clear that the effects of pitcher season via pitN do not exist in both Gerrit Cole’s slider and fastball pitching dynamics. The homogeneity in both Gerrit Cole’s slider and fastball pitching dynamics are established. Once again, we reiterate that the abundance and diversity of confirmed candidates of order-2 major factors reflect that this pitcher’s pitching dynamics is apparently free from the effects of heterogeneity induced by pitN.

## 5. Conclusions

In this paper, we demonstrate the merits of a CEDA-based selection protocol for collections of major factors as a brand new means of studying the dynamics underlying a single complex system as well as multiple complex systems in a collective fashion. We also show that the categorical nature of all features indeed is capable of revealing the governing principles underlying the system dynamics of interest, without any man-made structural modeling assumptions. In the real data analysis, we realistically see the complexity of Gerrit Cole’s pitching dynamics by using all selected collections of major factors of various orders. The major factor analysis reveals such complexity to a great extent and it reliably confirms the absence of any heterogeneity across the three considered MLB seasons. Such merits, capabilities and applicability support our CEDA paradigm as a fundamental approach for studying complex systems.

Our data-driven understanding is indeed supported by visible and explainable relational patterns found on the simple platform of contingency tables. Each contingency table of one or multiple major factors against the response variable will reveal conditional associative relations from the covariate categories to response categories. A collection of such pattern information will sustain and expand our knowledge about the complex systems under study. This fact is essential and important for data analysis in this Big Data era.

Furthermore, we once again emphasize that our CEDA computations work for all data types. This is an essential virtue of data analysis, since the categorical nature is present in all features of any data type. By employing such a categorical nature in data, the contingency table platform is natural, and so are information theoretic measurements. Consequently, the pattern formation brought out by conditional entropy and mutual information is authentic, and so is the understanding derived from these measurements.

By resolving the two real-world cases of pitching dynamics, we illustrate how to successfully implement the two criteria, [C1: confirmable] and [C2: irreplaceable], together with reliability checks. The CEDA-based methodology could prove critical when subjected to the finite sample phenomenon, even in the Big Data era. Finally, we reiterate the chief concept underlying our CEDA-based selection of major factors: “Let data’s categorical nature assemble freely and naturally to shed light on complexity, heterogeneity and homogeneity embedded within a collective of complex systems”.

## Figures and Tables

**Table 1:** Ten top-ranked CEs of feature sets across 1-feature to 4-feature settings. $Y=V_{1}+sin\left(2\pi \left(V_{2}+V_{3}\right)\right)+\frac{1}{10}N\left(0,1\right)$. CE of *Y* is 2.00773.

1-Feature	CE	2-Feature	CE	3-Feature	CE	4-Feature	CE
$V_{1}$	1.63636	$V_{1}\_V_{9}$	1.63607	$V_{1}\_V_{2}\_V_{3}$	1.07110	$V_{1}\_V_{2}\_V_{3}\_V_{8}$	1.05480
$V_{2}$	2.00768	$V_{1}\_V_{8}$	1.63609	$V_{1}\_V_{3}\_V_{9}$	1.63357	$V_{1}\_V_{2}\_V_{3}\_V_{9}$	1.05483
$V_{9}$	2.00768	$V_{1}\_V_{4}$	1.63610	$V_{1}\_V_{2}\_V_{9}$	1.63359	$V_{1}\_V_{2}\_V_{3}\_V_{4}$	1.05487
$V_{4}$	2.00769	$V_{1}\_V_{7}$	1.63610	$V_{1}\_V_{9}\_V_{10}$	1.63362	$V_{1}\_V_{2}\_V_{3}\_V_{10}$	1.05488
$V_{5}$	2.00769	$V_{1}\_V_{2}$	1.63610	$V_{1}\_V_{3}\_V_{8}$	1.63363	$V_{1}\_V_{2}\_V_{3}\_V_{6}$	1.05495
$V_{8}$	2.00769	$V_{1}\_V_{10}$	1.63611	$V_{1}\_V_{4}\_V_{7}$	1.63363	$V_{1}\_V_{2}\_V_{3}\_V_{5}$	1.05496
$V_{7}$	2.00769	$V_{1}\_V_{5}$	1.63611	$V_{1}\_V_{5}\_V_{7}$	1.63363	$V_{1}\_V_{2}\_V_{3}\_V_{7}$	1.05504
$V_{10}$	2.00770	$V_{1}\_V_{3}$	1.63612	$V_{1}\_V_{4}\_V_{5}$	1.63364	$V_{1}\_V_{4}\_V_{7}\_V_{9}$	1.61027
$V_{6}$	2.00770	$V_{1}\_V_{6}$	1.63612	$V_{1}\_V_{2}\_V_{8}$	1.63364	$V_{1}\_V_{5}\_V_{9}\_V_{10}$	1.61028
$V_{3}$	2.00770	$V_{2}\_V_{3}$	1.71415	$V_{1}\_V_{7}\_V_{9}$	1.63365	$V_{1}\_V_{3}\_V_{8}\_V_{9}$	1.61028

**Table 2:** Ten top-ranked “’successive CE drops” of feature sets across 1-feature to 4-feature settings. $Y=V_{1}+sin\left(2\pi \left(V_{2}+V_{3}\right)\right)+\frac{1}{10}N\left(0,1\right)$. CE of *Y* is 2.00773.

1-Feature	SCE-Drop	2-Feature	SCE-Drop	3-Feature	SCE-Drop	4-Feature	SCE-Drop
$V_{1}$	0.371372	$V_{2}\_V_{3}$	0.2935	$V_{1}\_V_{2}\_V_{3}$	0.5650	$V_{5}\_V_{7}\_V_{8}\_V_{10}$	0.0387
$V_{2}$	0.000051	$V_{5}\_V_{10}$	0.0004	$V_{2}\_V_{4}\_V_{9}$	0.0043	$V_{4}\_V_{8}\_V_{9}\_V_{10}$	0.0386
$V_{9}$	0.000048	$V_{3}\_V_{4}$	0.0004	$V_{8}\_V_{9}\_V_{10}$	0.0043	$V_{3}\_V_{4}\_V_{5}\_V_{9}$	0.0385
$V_{4}$	0.000040	$V_{3}\_V_{9}$	0.0004	$V_{3}\_V_{4}\_V_{5}$	0.0043	$V_{3}\_V_{6}\_V_{8}\_V_{9}$	0.0385
$V_{5}$	0.000040	$V_{8}\_V_{10}$	0.0004	$V_{4}\_V_{9}\_V_{10}$	0.0043	$V_{3}\_V_{4}\_V_{8}\_V_{10}$	0.0385
$V_{8}$	0.000038	$V_{7}\_V_{10}$	0.0004	$V_{3}\_V_{4}\_V_{6}$	0.0043	$V_{3}\_V_{5}\_V_{8}\_V_{9}$	0.0385
$V_{7}$	0.000036	$V_{4}\_V_{5}$	0.0004	$V_{5}\_V_{9}\_V_{10}$	0.0043	$V_{4}\_V_{5}\_V_{8}\_V_{9}$	0.0384
$V_{10}$	0.000035	$V_{5}\_V_{7}$	0.0004	$V_{7}\_V_{8}\_V_{10}$	0.0043	$V_{2}\_V_{4}\_V_{6}\_V_{7}$	0.0384
$V_{6}$	0.000032	$V_{2}\_V_{9}$	0.0004	$V_{5}\_V_{7}\_V_{10}$	0.0043	$V_{3}\_V_{5}\_V_{6}\_V_{7}$	0.0384
$V_{3}$	0.000027	$V_{8}\_V_{9}$	0.0004	$V_{6}\_V_{8}\_V_{9}$	0.0043	$V_{7}\_V_{8}\_V_{9}\_V_{10}$	0.0384

**Table 3:** The 8 top-ranked CEs across 1-feature to 4-feature settings with $Y=V_{2}+2V_{1}V_{7}+sin\left(2\pi \left(V_{3}+V_{6}\right)\right)+6V_{1}V_{3}V_{6}+sin\left(2\pi \left(V_{1}+V_{6}+V_{7}+V_{8}\right)\right)+\frac{1}{10}N\left(0,1\right)$. The CE of *Y* is 1.9945.

1 Feature	CE	2 Feature	CE	3 Feature	CE	4 Feature	CE
$V_{2}$	1.9366	$V_{3}\_V_{6}$	1.8543	$V_{1}\_V_{3}\_V_{6}$	1.6042	$V_{1}\_V_{3}\_V_{6}\_V_{7}$	1.4262
$V_{1}$	1.9407	$V_{1}\_V_{2}$	1.8678	$V_{2}\_V_{3}\_V_{6}$	1.7640	$V_{1}\_V_{2}\_V_{3}\_V_{6}$	1.4393
$V_{3}$	1.9673	$V_{1}\_V_{7}$	1.8812	$V_{3}\_V_{6}\_V_{7}$	1.7930	$V_{1}\_V_{3}\_V_{6}\_V_{8}$	1.5696
$V_{6}$	1.9675	$V_{2}\_V_{3}$	1.9031	$V_{1}\_V_{2}\_V_{7}$	1.7970	$V_{1}\_V_{3}\_V_{5}\_V_{6}$	1.5746
$V_{7}$	1.9918	$V_{2}\_V_{6}$	1.9033	$V_{1}\_V_{2}\_V_{6}$	1.8198	$V_{1}\_V_{3}\_V_{4}\_V_{6}$	1.5747
$V_{8}$	1.9945	$V_{1}\_V_{6}$	1.9052	$V_{1}\_V_{2}\_V_{3}$	1.8198	$V_{2}\_V_{3}\_V_{6}\_V_{7}$	1.6505
$V_{5}$	1.9945	$V_{1}\_V_{3}$	1.9054	$V_{1}\_V_{3}\_V_{7}$	1.8216	$V_{1}\_V_{2}\_V_{3}\_V_{7}$	1.6887
$V_{4}$	1.9945	$V_{2}\_V_{7}$	1.9326	$V_{1}\_V_{6}\_V_{7}$	1.8217	$V_{1}\_V_{2}\_V_{6}\_V_{7}$	1.6891

**Table 4:** The 8 top-ranked successive CE drops of feature sets across 1-feature to 4-feature settings. $Y=V_{2}+2V_{1}V_{7}+sin\left(2\pi \left(V_{3}+V_{6}\right)\right)+6V_{1}V_{3}V_{6}+sin\left(2\pi \left(V_{1}+V_{6}+V_{7}+V_{8}\right)\right)+\frac{1}{10}N\left(0,1\right)$. The CE of *Y* is 1.9945.

1 Feature	SCE-Drop	2 Feature	SCE-Drop	3 Feature	SCE-Drop	4 Feature	SCE-Drop
V2	0.057959	V3_V6	0.1130	V1_V3_V6	0.2501	V1_V3_V6_V7	0.1780
V1	0.053931	V1_V2	0.0688	V2_V3_V6	0.0903	V1_V2_V3_V6	0.1649
V3	0.027334	V1_V7	0.0594	V1_V2_V7	0.0709	V1_V6_V7_V8	0.1205
V6	0.027136	V1_V6	0.0355	V3_V6_V7	0.0613	V2_V3_V6_V7	0.1135
V7	0.002798	V1_V3	0.0353	V1_V3_V7	0.0596	V1_V2_V3_V7	0.1082
V8	0.000095	V2_V3	0.0336	V1_V6_V7	0.0596	V1_V2_V6_V7	0.1079
V5	0.000079	V2_V6	0.0333	V1_V2_V6	0.0481	V1_V3_V7_V8	0.0707
V4	0.000057	V3_V7	0.0084	V1_V2_V3	0.0480	V4_V5_V7_V8	0.0568

**Table 5:** Top 12 feature sets in selections for major factors in Gerrit Cole’s slider pitching dynamics with a 2D response variable $Y=(pfx_{X},pfx_{Z})$. The zero feature’s entropy is 4.6623.

1-Feature	CE	2-Feature	CE	3-Feature	CE	4-Feature	CE
aZ	2.6086	aX_aZ	0.6790	vY0_aX_aZ	0.2911	x0_aX_aY_aZ	0.0493
aX	2.6906	aX_spinD	1.5822	startSp_aX_aZ	0.2940	vY0_aX_aY_aZ	0.0518
spinD	3.1014	aZ_spinD	1.8457	x0_aX_aZ	0.3226	x0_vY0_aX_aZ	0.0534
spinR	4.1656	x0_aZ	1.9839	aX_aZ_spinR	0.3268	startSpeed_x0_aX_aZ	0.0551
vZ0	4.1742	aZ_spinR	1.9858	z0_aX_aZ	0.3287	z0_aX_aY_aZ	0.0557
x0	4.1798	z0_aZ	2.0045	aX_aY_aZ	0.3345	vX0_vY0_aX_aZ	0.0563
z0	4.1881	aY_aZ	2.0440	vZ0_aX_aZ	0.3468	startSp_aX_aY_aZ	0.0568
vY0	4.1953	vY0_aZ	2.0528	vX0_aX_aZ	0.3487	startSp_z0_aX_aZ	0.0570
startSp	4.1965	vX0_aZ	2.0590	aX_aZ_spinD	0.5539	z0_vY0_aX_aZ	0.0581
vX0	4.2031	startSp_aZ	2.0720	aX_aZ_pitN	0.5611	aX_aY_aZ_spinR	0.0619
aY	4.2224	vY0_aX	2.0798	x0_aY_aZ	0.7372	x0_aX_aZ_spinR	0.0624
pitN	4.3813	startSp_aX	2.0891	z0_aY_aZ	0.7430	vX0_aX_aY_aZ	0.0635

**Table 6:** Top 10 feature sets in selections for major factors in Gerrit Cole’s slider pitching dynamics with a 2D response variable Y=(aX,aZ). The zero feature’s entropy is 4.6652.

1-Feature	CE	2-Feature	CE	3-Feature	CE	4-Feature	CE
spinD	3.0958	vX0_spinD	2.2349	vZ0_aY_spinR	0.8346	x0_vZ0_aY_spinR	0.1287
spinR	4.1682	vZ0_spinD	2.2355	x0_vX0_spinD	0.8380	startSp_x0_vZ0_aY	0.1289
vZ0	4.1743	z0_spinD	2.3030	x0_vZ0_spinD	0.8396	z0_aY_spinD_spinR	0.1295
x0	4.1889	x0_spinD	2.3069	vZ0_aY_spinD	0.8406	x0_vY0_vZ0_aY	0.1299
z0	4.1897	spinD_spinR	2.3356	x0_aY_spinD	0.8517	z0_vZ0_aY_spinD	0.1308
vX0	4.1931	aY_spinD	2.3361	vX0_aY_spinD	0.8560	vZ0_aY_spinD_spinR	0.1316
vY0	4.2007	vY0_spinD	2.3651	z0_vZ0_spinD	0.8584	z0_vX0_aY_spinR	0.1337
startSp	4.2130	startSp_spinD	2.3788	z0_aY_spinD	0.8603	z0_vY0_vZ0_aY	0.1338
aY	4.2200	vX0_spinR	2.6619	z0_vZ0_aY	0.8683	x0_vY0_vZ0_spinR	0.1347
pitN	4.3800	vZ0_spinR	2.6624	x0_vX0_aY	0.8689	z0_vY0_vZ0_spinR	0.1357

**Table 7:** Top 12 feature sets in selections for major factors in Gerrit Cole’s fastball pitching dynamics with a 2D response variable $Y=(pfx_{X},pfx_{Z})$. The zero feature’s entropy is 4.6710.

1-Feature	CE	2-Feature	CE	3-Feature	CE	4-Feature	CE
spinD	3.1393	aX_aZ	1.6017	vY0_aX_aZ	0.7397	vX0_vY0_aX_aZ	0.2046
aX	3.1472	aZ_spinD	2.0316	startSp_aX_aZ	0.7472	startSp_vX0_aX_aZ	0.2057
aZ	3.3218	aX_spinD	2.1719	vZ0_aX_aZ	1.0378	startSp_vZ0_aX_aZ	0.2080
spinR	4.2977	vY0_aX	2.5007	vX0_aX_aZ	1.0405	vY0_aX_aY_aZ	0.2123
aY	4.3491	startSp_aX	2.5092	x0_aX_aZ	1.0607	vY0_vZ0_aX_aZ	0.2126
x0	4.3706	vY0_aZ	2.5849	z0_aX_aZ	1.0709	x0_vY0_aX_aZ	0.2129
z0	4.3743	startSp_aZ	2.5961	aX_aY_aZ	1.0783	startSp_aX_aY_aZ	0.2160
pitN	4.4082	aX_spinR	2.6144	aX_aZ_spinR	1.0846	z0_vY0_aX_aZ	0.2177
vZ0	4.4247	aX_aY	2.6322	vY0_aZ_spinD	1.1731	startSp_x0_aX_aZ	0.2227
vX0	4.4597	vZ0_spinD	2.6681	startSp_aZ_spinD	1.1774	startSp_z0_aX_aZ	0.2236
vY0	4.4761	aY_spinD	2.6700	vY0_vZ0_aX	1.3215	startSp_aX_aZ_spinR	0.2635
startSp	4.4860	x0_aX	2.6725	startSp_vZ0_aX	1.3295	vY0_aX_aZ_spinR	0.2637

**Table 8:** Top 10 feature sets in selections for major factors in Gerrit Cole’s fastball pitching dynamics with a 2D response variable Y=(ax,az). The zero feature’s entropy is 4.6913.

1-Feature	CE	2-Feature	CE	3-Feature	CE	4-Feature	CE
spinD	3.1776	aY_spinD	2.6820	vZ0_aY_spinD	1.4493	startSp_x0_vX0_vZ0	0.3130
spinR	4.2681	vZ0_spinD	2.6894	vY0_vZ0_spinD	1.4529	startSp_z0_vX0_vZ0	0.3142
aY	4.3476	spinD_spinR	2.6915	startSpeed_vZ0_spinD	1.4591	x0_vX0_vY0_vZ0	0.3150
x0	4.3833	vX0_spinD	2.7231	startSpeed_vX0_spinD	1.4784	startSp_x0_vZ0_aY	0.3164
z0	4.3851	startSp_spinD	2.7502	vX0_vY0_spinD	1.4838	z0_vX0_vY0_vZ0	0.3211
pitN	4.4106	vY0_spinD	2.7556	vX0_aY_spinD	1.4843	x0_vX0_vZ0_aY	0.3213
vZ0	4.4387	x0_spinD	2.7664	vX0_vZ0_spinD	1.4849	x0_vY0_vZ0_aY	0.3247
vY0	4.4532	z0_spinD	2.8026	vZ0_spinD_spinR	1.4969	z0_vX0_vZ0_aY	0.3255
startSp	4.4548	spinD_pitN	2.9914	startSp_x0_vX0	1.5085	vX0_vY0_vZ0_aY	0.3295
vX0	4.4712	vZ0_spinR	3.2976	x0_vZ0_aY	1.5097	startSp_vX0_vZ0_aY	0.3318

**Table 9:** Comparison of major factors of Gerrit Cole’s slider and fastball with respect to $Y=(pfx_{X},pfx_{Z})$ and Y=(aX,aZ).

Pitch Type	Y	Order-1 MF	Order-2 MF	Alternative MF	Alternative MF
slider	$\left(pfx_{X},pfx_{Z}\right)$	aZ,x0	none confirmed	{aZ,spinR}	{aZ,z0}
slider	(aX,aZ)	spinD,vX0	none confirmed	{spinD,vZ0}	{spinD,z0)}
fastball	$\left(pfx_{X},pfx_{Z}\right)$	aX	(aZ,vY0)	{aX,aZ}	{aX,vY0}
fastball	(aX,aZ)	spinD,aY	none confirmed	{spinD,vZ0}	{spinD,spinR}

## Data Availability

The pitching data are available in PITCHf/x database belonging to Major League Baseball via http://gd2.mlb.com/components/game/mlb (accessed on 8 May 2021).
